# Pathotyping and Antibiotic Resistance Profiling of *Escherichia coli* Isolates from Children with Acute Diarrhea in Amatole District Municipality of Eastern Cape, South Africa

**DOI:** 10.1155/2020/4250165

**Published:** 2020-11-19

**Authors:** S. A. Omolajaiye, K. O. Afolabi, B. C. Iweriebor

**Affiliations:** ^1^Department of Surgery, Ladysmith Regional Hospital, 36 Malcom Road, Ladysmith, 3370 KwaZulunatal, South Africa; ^2^Department of Biological Sciences, Anchor University, Lagos, Nigeria; ^3^Department of Biology, Sefako Makgatho Health Sciences University, Ga-Rankuwa, Pretoria, South Africa

## Abstract

**Background:**

Diarrhea has been reported as the leading cause of childhood mortality and morbidity globally but with disproportionate impacts in developing nations. Among bacterial etiologic agents of diarrhea, diarrheagenic *Escherichia coli* is the main cause of the disease among children under the age of 5 years. This study is aimed at determining the prevalence and antibiogram pattern of diarrheagenic Escherichia *coli* (DEC) pathotypes associated with diarrhea cases in the study area.

**Methods:**

A total of 120 presumptive isolates of *E. coli* were obtained from diarrheal stool samples from male and female patients below 12 years of age using chromogenic agar. Confirmation of the isolates and screening for virulence genes were determined by polymerase chain reaction (PCR) while antimicrobial susceptibility testing was performed using the disk diffusion method. The presence of antibiotic resistance genes to chloramphenicol and tetracycline among the confirmed isolates was also profiled by PCR based on the observed phenotypic resistance pattern.

**Results:**

Of the 120 presumptive isolates, 88.3% (106/120) were confirmed as *E. coli* through PCR. The molecular pathotyping of the confirmed isolates showed their distribution as 41% (43/106) of diffusely adhering *E. coli* (DAEC), 17% (18/106) of enterohemorrhagic *E. coli* (EHEC), 17% (18/106) of enteropathogenic *E. coli* (EPEC), and 10% (11/106) of enteroinvasive *E. coli* (EIEC), while enteroaggregative *E. coli* (EAEC) and enterotoxigenic *E. coli* (ETEC) were not detected, and the remaining 15% did not belong to any pathotype. Notably, high resistance of the isolates to commonly used antimicrobials was observed as follows: ampicillin (98%), chloramphenicol (94%), trimethoprim-sulfamethoxazole (96%), and tetracycline (90.6%), while a relatively low number of the confirmed isolates were resistant to ciprofloxacin (45%) and imipenem (36%). In addition, 94% of the isolates that exhibited phenotypic resistance against chloramphenicol harbored the *cat*A1 resistance gene while 89% that showed resistance to tetracycline had *tet*A genes.

**Conclusions:**

These findings showed that DEC could be considered as the leading etiologic bacterial agent responsible for diarrhea in the study community, and the observable high degree of resistance of the isolates to antimicrobial agents is of huge significance, calling for stakeholders to adopt and consolidate the existing antimicrobial stewardship scheme of the government, in order to ensure an uncompromised public health.

## 1. Introduction

Diarrhea has been described as one of the leading causes of illness and death in children under the age of 5 years, predominantly in underdeveloped countries [1]. However, investigations have confirmed that almost all cases of deaths due to diarrhea could be prevented [2]. In 2010, out of about 5 million deaths recorded worldwide due to infectious diseases in children under the age of five, and diarrheal diseases accounted for over 10% of deaths, ranking second after pneumonia [2]. Nevertheless, there has been a reduction in the childhood death rates worldwide in recent years as a result of oral rehydration therapy. The prevalence of this disease has been clearly associated with contributory factors such as untimely weaning of children from breast feeding, drinking of unsafe water, encouraging bottle-feeding, and malnutrition [3].

A diversity of pathogens including viruses, bacteria, and parasites are causative agents of infectious diarrhea [4]. Various studies have confirmed that, of the bacterial pathogens, diarrheagenic *E. coli* (DEC) has been described as an incriminating pathogen of acute infectious diarrhea in children in developing countries [5]. The Global Burden Disease Report of the WHO described diarrhea as the second most common cause of mortality in children under five with DEC being responsible for most of the cases [6]. In developing nations, a high number of children suffer over a dozen episodes of diarrhea in their first year. Children who suffer continual and persistent diarrhea are likely to experience some psychosocial issues and growth retardation as well as loss of cognitive functions [7].

Diarrheal diseases have been a contributing factor in child undernourishment and deterioration in growth and development. Besides other typical infections, diarrhea caused by *E. coli* may be even more harmful than rotavirus infections [8]. Children are more prone to repeated diarrheal episodes caused by diverse strains of diarrheagenic *E. coli* as a result of immature immune systems. There are two classes of *E. coli* pathogenic strains which are DEC and the extra intestinal pathogenic *E. coli* (ExPEC) that includes UPEC (related to UTI), SEPEC (related to sepsis), and NEMEC (related to neonatal meningitis) [9, 10]. There are six fundamentally recognized strains of diarrheagenic *E. coli* (DEC) that are related to diarrhea based on their different clinical features, virulence factors, and serotypes grouping, and they are enteroaggregative *E. coli* (EAEC), enteroinvasive *E. coli* (EIEC), enterohemorrhagic *E. coli* (EHEC), enteropathogenic *E. coli* (EPEC), diffusely adherent *E. coli* (DAEC), and enterotoxigenic *E. coli* (ETEC) [11]. Out of all diarrheal pathogens, diarrheagenic *E. coli* is known as the leading agent of diarrheal diseases. In a previous study carried out by Ochi et al. [12], diarrheagenic *E. coli* was detected as an etiologic agent in 20% of diarrheal specimens, 34% of which were from children under 5 years old. In a study based on children with diarrhea in Mozambique, the prevalence of diarrheagenic *E. coli* was 42%, and rotavirus had a prevalence of 18%, while parasites accounted for 38% [13]. *E. coli* is a normal flora of the gastrointestinal tract (GIT) of humans and other mammals, where it contributes greatly to metabolic activities and production of some GIT vitamins. The significance of avirulent strains of *E. coli* has been exploited widely in recombinant DNA technology. However, when it acquires certain pathogenic factors, *E. coli* can become a highly virulent pathogen [14]. *E. coli* pathogenic tendencies lie in its ability to express genetic flexibility through the acquisition and/or transfer of virulence genes by both vertical and horizontal gene transfer mechanisms [15].

Apart from the virulence gene acquisition by *E. coli* strains, there have been myriads of cases of antibiotics resistance gene possession by the organism found in both clinical, animal, and environmental samples [16, 17]. The high rate of incidences of bacterial resistance to commonly used antimicrobial agents (tetracycline and chloramphenicol) in treating diarrheal diseases is a concern and a major threat to human health; hence, this should be properly monitored in order to establish the extent of their spread and inform necessary mitigating strategy. The Amatole District Municipality is rural with insufficient portable water supply coupled with high HIV prevalence. This compromised health condition along with lack of access to portable water exposes the population to high prevalence of gastroenteritis. Hence, we hereby report the prevalence, antibiogram, and putative virulence gene profiles of *E. coli* pathotypes isolated from stool samples of diarrheal patients from hospitals in the Eastern Cape Province, South Africa.

## 2. Materials and Methods

### 2.1. Ethics and Informed Consent

Ethical clearance to carry out this research was obtained from the University of Fort Hare research ethics committee (ref no.: OKO011SOMO01) and the Eastern Cape Department of Health (ref no.: EC-20146RP10-487), while informed consent was obtained from each patient before sampling and strict confidentiality of the participated patients was maintained.

### 2.2. Inclusion and Exclusion Criteria

In patients' recruitment, only those with symptoms of diarrhea as defined by WHO [18] and who were not on any antibiotic treatment were recruited into the study. Those who had diarrhea but were on antibiotics treatment were excluded. None diarrhea participants were not recruited into the study. All study participants were under the age of 12 years, and sampling was a once-off prospective sampling.

### 2.3. Sampling

Between March, 2015 and May, 2017, 95 diarrheal stool samples were collected from diarrhea patients in some private and public medical facilities in the Amatole District Municipality. Patients who were admitted into the health facilities or those who came in as outpatients with complaints of diarrhea were recruited into the study at the once-off sampling procedure. Only patients with symptoms of diarrhea which is defined as passage of three or more loose or liquid stools per day or frequent passage than is normal for the individual were recruited into the study. Fresh samples were obtained directly from the patients who had just passed watery stool at the time of sampling and from the anorectal cavity of some patients who were still passing watery stools but not at the time of sample collection using sterile swab sticks. Patients who had commenced treatment or had taken antibiotics in the last one month were excluded from the study. The samples were taken from patients below 12 years of age irrespective of sex and race. After collection, the samples were conveyed on ice packs to the Applied and Environmental Microbiology Research Group (AEMREG) laboratory at the University of Fort Hare, Alice, for analysis within 24 hours of collection.

### 2.4. Isolation of Presumptive *E. coli* Isolates

The bacteriological analyses of collected samples were performed using standard methods as follows: diarrheal samples (on swab sticks) were streaked directly on *E. coli* chromogenic agar (Sigma-Aldrich-73009). The plates were incubated at 37°C for 24 h, and presumptive isolates (2-3 distinct colonies per plate) which were blue or purple colored were picked with sterile wire loop and inoculated into 2 mL Tryptone Soya Broth (TSB) and incubated at 37°C for 24 h. Thereafter, the culture was used to prepare 30% glycerol stocks and stored at -80°C for further analyses.

### 2.5. DNA Extraction and Molecular Confirmation of Presumptive *E. coli* Isolates

Bacterial genomic DNA extraction using the boiling method and the molecular confirmation of the presumptive *E. coli* isolates were done as described by Iwu et al. [19]. Verification of PCR amplification products was performed in 1.5% agarose gel stained with ethidium bromide and electrophoresed at 120 V for 45 min using 0.5 × Tris-borate-EDTA (TBE) buffer and then viewed in UV transilluminator (ALLIANCE 4.7).

### 2.6. Delineation of *E. coli* Pathotypes among Study Isolates

Delineation of the confirmed *E. coli* isolates into respective pathotypes was done by PCR using specific primer pairs targeting the relevant virulence genes of EHEC, EPEC, ETEC, EIEC, EAEC, and DAEC pathotypes as listed in Table 1. The reaction mixture (25 *μ*L) contained 1 *μ*L each of 10 pmol of both forward and reverse specific primer pairs, 12.5 *μ*L of PCR master mix (New England Biolabs-NEB), 5.5 *μ*L of nuclease free water, and 5.0 *μ*L of DNA template. The cycling conditions were as follows: 94°C initial denaturation for 5 min, followed by 35 cycles at 94°C in 60 sec, annealing temperature as shown in Table 2 for respective primers for 30 sec, 72°C (extension) for 60 sec, and a final elongation step at 72°C for 5 min. PCR products were verified by electrophoresis in 1.5% agarose gel stained with ethidium bromide, visualisation, and documentation were done using an Alliance 4.7 transilluminator. *E. coli* ATCC 25922 was used as a positive control in the confirmation of the presumptive isolates.

### 2.7. Antibiotic Susceptibility Profiling

The antibiotic susceptibility profiles of the confirmed 106 *E. coli* isolates were determined according to Clinical and Laboratory Standard Institute (CLSI) [23] guidelines on Mueller-Hinton agar. The confirmed isolates in glycerol stocks were resuscitated in TSB (tryptone soya broth) and incubated at 37°C for 24 h. The TSB culture matching 0.5 McFarland standards was evenly inoculated onto Mueller-Hinton agar with sterile swab sticks, allowed to dry for 10 min, and antibiotic discs were dispensed using an antibiotic disc dispenser. Empiric antibiotics used in the treatment of diarrhea were employed in the susceptibility testing. Each positive sample was tested against the following 12 antibiotics belonging to the following antibiotic groups: (i) *β*-lactam (ampicillin (10 *μ*g), (ii) cephalosporin: cefotaxime (30 *μ*g) and cefuroxime (30 *μ*g), (iii) chloramphenicol: chloramphenicol (30 *μ*g), (iv) fluoroquinolone: norfloxacin (10 *μ*g) and ciprofloxacin (5 *μ*g), (v) sulfonamides: trimethoprim-sulfamethoxazole (25 *μ*g), (vi) carbapenem: imipenem (10 *μ*g), (vii) macrolide:-erythromycin (15 *μ*g), (viii) aminoglycoside: gentamicin (10 *μ*g), and (ix) tetracyclines: tetracycline (30 *μ*g) and doxycycline (30 *μ*g). Thereafter, the plates were incubated at 37°C for 24 h and read for sensitivity. The antibiotics chosen are typically used for the treatment of diarrheal diseases caused by *E. coli.*

### 2.8. Screening for Antimicrobial Resistance Genes

Chloramphenicol and tetracycline resistance genes among the isolates were assessed based on the observed phenotypic resistance patterns by PCR using specific primers targeting *cat*A1 and *tet*A resistance genes as presented in Table 2. PCR was performed in a 25 *μ*L reaction mixture containing 1 *μ*L each of both forward and reverse specific primer pairs, 12.5 *μ*L of PCR master mix (New England Biolabs-NEB), 5.5 *μ*L of nuclease free water, and 5.0 *μ*L of DNA template. The cycling conditions were as follows: 94°C initial denaturation for 5 min, followed by 35 cycles of 94°C in 60 sec, 53°C and 52°C (annealing temperature for *cat*A1 and *tet*A, respectively) for 30 sec, 72°C (extension) for 60 sec, and a final elongation step at 72°C for 5 min. Amplification of PCR product was verified by electrophoresis in a 1.5% agarose gel stained with ethidium bromide, visualized and documented in an Alliance 4.7 transilluminator. *E. coli* ATCC 25922 was used a negative control in antibiotic resistance determinant testing.

## 3. Results

### 3.1. Isolation and Confirmation of *E. coli*

A total of 120 presumptive isolates of *E. coli* were obtained from the diarrheal stool samples collected from both outpatients and inpatients attending or admitted in private and public hospitals in the Amatole District Municipality of Eastern Cape Province. A total of 95 stool samples were collected from the study participants whose ages ranged from less than a year to 12 years. The basic information about the patients and the seasonal distribution of diarrhea in the study period are shown in Table 3.

**Figure 1:**
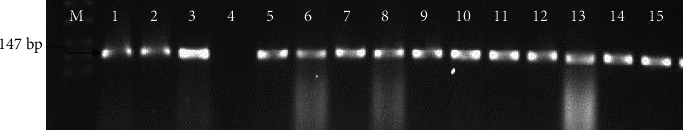
Confirmation of the presumptive *E. coli* isolates through the amplification of *uid*A genes (147 bp). Lane M: molecular weight marker (50 bp); lanes 1 to 3: positive control (*E. coli* ATCC 25922); lane 4: nuclease free water as negative control; lanes 5 to 15: representative positive isolates.

Out of the 120 presumptive isolates, 106 were confirmed as *E. coli* through PCR (Figure 1), representing 88.3% of the total presumptive isolates.

### 3.2. Delineation of the PCR-Confirmed Isolates into Pathotypes

**Figure 2:**
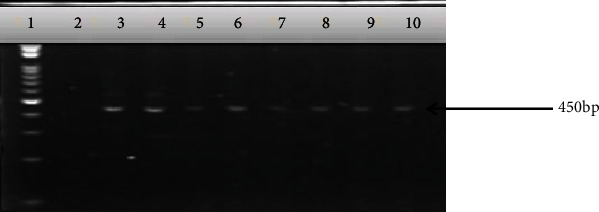
Gel electrophoresis of PCR products amplified with the e*ae*A primers for the detection of enterohemorrhagic *E. coli*. Lane 1: DNA ladder 100 bp; lane 2: nuclease-free water as negative control; lanes 3-11: enterohemorrhagic *E. coli.*

**Figure 3:**
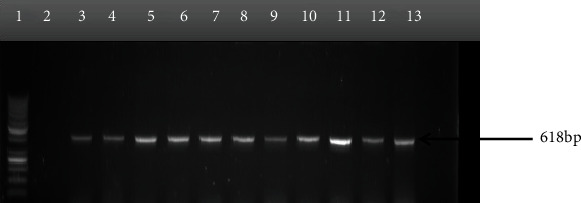
A gel electrophoresis of PCR products amplified with *vir* primers for the detection of enteroinvasive *E. coli* (EIEC). Lane 1: 100 bp DNA ladder; lane 2: nuclease-free water as negative control; lanes 3 to 13: *E. coli* isolates.

**Figure 4:**
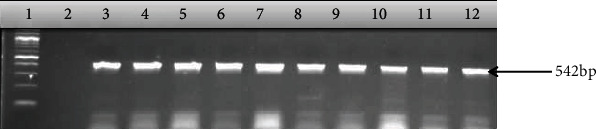
Gel electrophoresis of PCR products amplified with thye *daaE* gene for the detection of diffusely adherent *E. coli*. Lane 1: 100 bp DNA ladder; lane 2: nuclease-free water as negative control; lanes 3-12: diffusely adherent *E. coli.*

**Figure 5:**
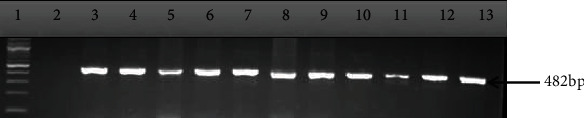
Gel electrophoresis of PCR products amplified with *eae* (intimin) primers for the detection of enteropathogenic *E. coli*. Lane 1: 100 bp DNA ladder; lane 2: nuclease-free water negative control; lanes 3-13: enteropathogenic *E. coli*.

The outcome of molecular delineation of the confirmed isolates into pathotypes showed that 90 out of the 106 isolates possessed virulence genes which form the basis of their pathotyping as shown in Table 4 and Figures 2–5. The pathotype prevalence were DAEC (41%), EHEC (17%), EPEC (17%), and EIEC (10%), representing 85% of the confirmed isolates, while EAEC and ETEC were not detected.

### 3.3. Antibiogram Profiles and Prevalence of Antibiotic Resistance Genes among the Confirmed *E. coli* Isolates

The antibiotic susceptibility profiles of all the confirmed *E. coli* isolates are summarized in Table 5. Notably, a very high resistance to commonly used antibiotics such as ampicillin (98%), chloramphenicol (94%), trimethoprim-sulfamethoxazole (96%), and tetracycline (91%) was observed, while relatively lower values were recorded for ciprofloxacin (45%) and imipenem (36%). Furthermore, as expected due to observable phenotypically high resistance of the isolates to the studied chemotherapeutic agents by the conventional susceptibility profiling, high prevalence of 94% and 89% was observed in the isolates for the resistance genes *cat*A1 and *tet*A for chloramphenicol and tetracycline, respectively, which undoubtedly confer the high level resistance of the isolates to the respective antibiotics.

## 4. Discussion

In this study, the prevalence and the antibiogram patterns of diarrheagenic *E. coli* (DEC) isolated from patients attending some hospitals in the Amatole District Municipality of Eastern Cape Province, South Africa, were investigated. Related findings from previous studies in the study area have shown the occurrence of various *E. coli* strains including the O157 : H7, which is the etiologic agent of enteric infections such as bloody diarrhea, from other sources including surface water, waste water effluents, swine, cattle, and fresh cow milk, representing the environmental and animal sources. In a study, Iweriebor et al. [25] reported about 32% prevalence of *E. coli* O157:H7 in cattle from two dairy farms situated in one of the local municipalities in the district, when faecal samples numbering 400 were screened from the cattle population of 920. Subsequently, Msolo et al. [26] asserted the possibility of dairy and dairy products in the municipality to be a potent reservoir of *E. coli* O157:H7 with its prevalence of 11% in samples of raw milk, cattle udder, milking machine, and hands of dairy workers. Similarly, some intestinal pathogenic *E. coli* (InPEC) strains were equally detected in treated final effluents meant to be discharged into the receiving water body in the area, with the prevalence of 1.4% recorded for ETEC and 7.6% for both EPEC and EAEC [27]. More recently, Igwaran et al. [28] equally reported 8.1% prevalence for another InPEC, namely, DAEC pathotype from the final effluent of waste water treatment plants (WWTPs) of two major towns within the district municipality. This current study is undoubtedly of huge relevance being the first to give insight into the prevalence of diarreagenic *E. coli* in clinical samples of human origin obtained from patients at some health facilities in the district municipality, thereby confirming the role of the organism as major etiologic agent in diarrhea cases in the study area.

In this study, 106 isolates were confirmed to be DEC, which is 88.3% of the total isolates obtained from culture prior to molecular confirmation. This result is significant and showed that DEC is a leading bacterial agent causing diarrhea in the study community, thereby solidly supports various epidemiological findings that have previously confirmed DEC as the most frequently isolated enteropathogenic bacteria from diarrhea cases globally [29–31]. However, a number of studies have reported some variations. For instance, in Tunisia and New Caledonia, *Salmonella* spp. was reported as the most frequently identified enteropathogens [32], while *Campylobacter jejuni* was described as the leading enteropathogen in adults in Sweden [33]. Nevertheless, in South Africa and some other southern African countries, DEC remains the leading bacterial agent causing diarrheal diseases [34]. The notable geographical differences in prevalence of the organism could be a function of many factors including size of population studied and various microbiological techniques employed in the studies. However, in the overall, and as it is supported by findings from this work, DEC strains have been frequently isolated and implicated in cases of diarrhea from the most developing and developed countries including India [35], Nigeria [36], Thailand [37], and Republic of Korea [30].

The most prevalent pathotype of DEC encountered in this study was diffusely adhering *E. coli* (DAEC) (41%), followed by enterohemorrhagic *E. coli* (EHEC) (17%), enteropathogenic *E. coli* (EPEC) (17%), and enteroinvasive *E. coli* (EIEC) (10%), while no enteroaggregative *E. coli* (EAEC) or enterotoxigenic *E.coli* (ETEC) were detected. This is in agreement with the reports from Maputo, Mozambique, in which DAEC frequency was higher than that of EPEC, ETEC, EIEC, or EAEC in diarrheal stool samples [13]. Studies have shown that DAEC is widespread and may be more prevalent in HIV-positive patients [6, 31, 38]. Interestingly, in a human challenge study, it was discovered that some DAEC strains did not produce diarrhea in healthy individuals; however, since the category of bacteria is heterogenous, being unable to cause diarrhea in older people does not denote an absence of virulence in more susceptible persons [39].

As earlier mentioned, the relatively higher prevalence of 17% was also recorded for EPEC in this study, and this corroborates findings from various studies in many parts of Africa and worldwide [13, 40, 41]. In South Africa also, a study conducted in Western Cape identified EPEC as the most prevailing cause of diarrhea during childhood [42]. However, there has been a decline in relevance of EPEC as a pathogen in some published articles. This could be associated with the practices such as breast feeding which has been reported to greatly prevent diarrhea caused by EPEC [40]. That is, the significant decline in the prevalence of diarrhea caused by EPEC could be linked to the UNICEF/WHO 0-6 month uninterrupted breastfeeding advocacy [2]. However, it has become evident that EPEC is the prevailing cause of diarrhea in people with HIV [31]. In an inclusive study on bacterial enteropathogens carried out among hospitalized patients with diarrhea in all age groups in India, EPEC was found to be the prevailing pathotype of DEC [43].

In the same vein, the prevalence of 17% that was also recorded for EHEC in this study is of huge significance considering the havoc that outbreaks of the *E coli* strain previously rendered in Southern Africa countries and beyond. EHEC was first documented in South Africa in 1990 in a periodic episode of hemorrhagic uremic syndrome caused by EHEC O157 : H7 [44]. Three years later, an outbreak of EHEC occurred in a sugar plantation in Swaziland which resulted in the death of approximately 2,000 people [45]. Also, in another part of Africa, precisely Central African Republic, Germani et al. [33] reported an EHEC upsurge in which 108 people presenting with bloody diarrhea were admitted to the hospital, out of which the death of 4 infected individuals was recorded. Furthermore in 2004, an outbreak of over 300 cases of diarrhea with bloody stool was reported in some parts of Ngoila town in Cameroon [46]. EHEC diarrhea epidemics have also been described in North America and Western Europe due to the consumption of undercooked beef, as well as from eating vegetables fertilized with animal manure [47]. In essence, it has been asserted that sporadic and epidemic EHEC infections occur both in developing and developed countries of the world, and that routine surveillance is imperative in identifying outbreaks and help in determining various reservoirs and transmission routes [29].

The EIEC in this study was detected in 10% of the total confirmed isolates. However, no EIEC was identified in a study conducted in Gabon, while reports from studies carried out in Kenya, Mozambique, Ghana, and Nigeria also identified a small number of EIEC pathotypes [48]. The prevalence of this pathotype of *E. coli* has been rarely reported as a diarrheal agent in teenagers and adults but in Ecuador, reports from one study found EIEC to be a leading cause of infectious diarrhea in all age groups [49]. Most other studies also rarely report this pathotype in adults of all ages. In this study, ETEC and EAEC, which are predominantly known to be responsible for travellers' diarrhea, [50] were not detected. The probable reason for not detecting EAEC could be attributed to the primers used because the PCR multiplex assay by Vidal et al. [22] identifies AAF/II-positive EAEC strains, and not all EAEC adhere by virtue of AAF fimbriae.

The conspicuously increased rate of antimicrobial resistance of some strains of diarrheagenic bacterial pathogens across the globe is quite alarming, more significantly in less developed regions [51]. The indiscriminate use of antibiotics in the treatment of diarrheal diseases has been noted as a cogent reason for the high rate of antimicrobial resistance [52]. DEC belongs to the group of GIT commensal bacteria which have been reported to be the principal determinant of resistance genes for pathogenic bacteria [53], and *E. coli* has often been used as an important index in surveying the critical selection of antimicrobial agents and determination of genes responsible for resistance [54]. Previously, some investigations have stipulated that most DEC strains have exhibited antibiotic resistance to at least ampicillin, sulfonamide, or cotrimoxazole [42]. Certain antibiotics which no longer cure diarrhea are still constantly prescribed to treat infectious diarrhea due to their accessibility and low cost [6, 55]. The most frequently used antibiotics in treating diarrhea are tetracycline, cotrimoxazole, and ampicillin [56].

In this study, a high rate of DEC antimicrobial resistance to frequently used antibiotics such as ampicillin (98%), tetracycline (91%), chloramphenicol (94%), and trimethoprim-sulfamethoxazole (96%) was noticed. This finding is similar to some reports from various studies in children from Bolivia, Peru, Mozambique, Vietnam, Mexico, Argentina, and Tanzania, which observed over 70% prevalence of ampicillin resistance in DEC isolates identified [57]. In another study conducted by Nguyen et al. [56] in Honoi, Vietnam, DEC pathotypes exhibited different degrees of resistance to ampicillin, chloramphenicol, and trimethoprim-sulfamethoxazole which ranged from 19.1% to 86.4%. In a similar study in Egypt, the prevalence of resistance among some DEC isolates ranged from 24.2% to 68.2% for ampicillin-sulbactam, ampicillin, and trimethoprim-sulfamethoxazole [58]. Bouzari et al. [55] reported a high prevalence of resistance against trimethoprim-sulfamethoxazole, tetracycline, and chloramphenicol in DEC strains isolated from Tehran, Iran, which is similar to the findings of this study. Several studies have documented the common occurrence of multidrug-resistant DEC pathotypes which could possibly be attributed to environmental acquisition of resistant genes, transmission of pathogens among humans of all age groups or by zoonosis [59], and occasionally as a result of indiscriminate use of different antibiotics in the management of infectious diarrhea [52].

In this study, resistance to imipenem, ciprofloxacin, and norfloxacin (36%, 45%, and 57, respectively) was relatively lower than other studies. This corroborates a study that recommended fluoroquinolones as first-line drugs to treat diarrhea [51]. In a bid to screen for the presence of antibiotic resistance genes in DEC strains obtained in this study, isolates that were resistant to chloramphenicol and tetracycline were preferentially selected and profiled for tetracycline and chloramphenicol resistance determinants. Findings revealed that 94% and 89% of the isolates harbored *cat*A1 and *tet*A genes, respectively. This is much comparable to 91% (*cat*A1) and 93% (*tet*A) obtained in a study conducted at North West England by Ahmed et al. [60], and it further justifies the observable high level resistance of the isolates to the respective antibiotics.

## 5. Conclusions

The relatively high prevalence of DEC in this study is remarkable, and it highlights the fact that the organism is a significant agent of infectious diarrhea and could be the leading cause of gastroenteritis in the Eastern Cape. Furthermore, the notable high resistance of the isolates to commonly used antibiotics in the treatment of diarrhea is of huge concern, and it emphasizes the need for more proactive measures by the stakeholders in curbing the menace. However, observable phenotypically high resistance of the isolates to the studied chemotherapeutic agents by the conventional susceptibility profiling should give clues to health practitioners in the area on the best antibiotics of choice in treating diarrhea as a result of DEC infections.

## Figures and Tables

**Table 1:** Primer sequences used for the molecular confirmation of *E. coli* isolates and identification of its pathotypes.

Target strain/gene	Primer sequence 5′-3′	Annealing temp (°C)	Band size (bp)	References
*E. coli* (*uid*A)	AAAACGGCAAGAAAAAGCAG ACGCGTGGTTACAGTCTTGCG TCA ATG CAG TTC CGT TAT CAG TT	50 55	147 450	[20] [21]
*eae*A (EHEC)	GTA AAG TCC GTT ACC CCA ACC TG GCACACGGAGCTCCTCAGTCTCC	56	218	[21]
*lt* (ETEC)	TTCATCCTTTCAATGGCTTT AGCTCAGGCAATGAAACTTTGAC	54	618	[22]
*vir* (EIEC)	TGGGCTTGATATTCCGATAAGTC TCAATGCAGTTCCGTTATCAGTT	55	482	[21]
*eae* (EPEC)	GTAAAGTCCGTTACCCCAACCTG CACAGGCAACTGAAATAAGTCTGG	56	378	[22]
*aaf*II (EAEC)	ATTCCCATGATGTCAAGCACTTC GAACGTTGGTTAATGTGGGGTAA	56	542	[22]
*daa*E (DAEC)	TATTCACCGGTCGGTTATCAGT			

**Table 2:** Primers for the screening of various resistant *E*. *coli* isolates for the presence of antimicrobial resistance genes.

Antimicrobial agents	Resistance gene	Primer sequence (5′-3′)	Size (bp)	Annealing temp (°C)	References
Chloramphenicol	*cat*A1	AGTTGCTCAATGTACCTATAACC	547	53	[16]
		TTGTAATTCATTAAGCATTCTGCC			
Tetracycline	*tet*A	GGTTCACTCGAACGACGTCA	577	52	[24]
		CTGTAAGACAAGTTGCATGA			

**Table 3:** Background information about the 95 study patients with diarrhea.

Attribute	Number (%)
Age	
<1 year	15 (15.79%)
>1-3 years	60 (63.16%)
>3-5 years	10 (10.53%)
>5-10 years	7 (7.38%)
>10 years	3 (3.16%)
Sex	
Male	45 (47.37%)
Female	50 (52.63%)
Season	
Autumn/fall	13 (13.6%)
Winter	2 (2.1%)
Spring	20 (21.05%)
Summer	60 (63.2%)

**Table 4:** The pathotypes prevalence among confirmed 106 isolates according to patient's age group.

*E.coil* pathotypes	Target gene	Prevalence (%)	0-11 months	>1-3 yrs	>3-5 yrs	>5-10 yrs	>10 yrs
DAEC	*daa*E	43 (41%)	16 (37.21%)	23 (53.49%)	3 (7%)	1 (2.33%)	
EHEC	*eae*A	18 (17%)		2 (11.11%)	5 (27.78%)	1 (5.56%)	10 (55.56%)
EIEC	*Vir*	11 (10%)			8 (72.73%)	2 (18.2%)	1 (9.1%)
EPEC	*Eae*	18 (17%)	8 (44.44%)	6 (33.33%)	3 (16.67%)	1 (5.6%)	

**Table 5:** Resistance pattern of the confirmed *E. coli* isolates to commonly used antibiotics.

Antibiotics	Resistance (%)
Ampicillin	98
Cefotaxime	96
Cefuroxime	89
Chloramphenicol	94
Trimethoprim-sulfamethoxazole	96
Imipenem	36
Erythromycin	92
Gentamicin	66
Tetracycline	91
Ciprofloxacin	45
Doxycycline	94
Norfloxacin	57

## Data Availability

All data and materials are presented in the manuscript.

## Abbreviations

PCR: Polymerase chain reaction
DAEC: Diffusely adhering *E. coli*
EHEC: Enterohemorrhagic *E. coli*
EIEC: Enteroinvasive *E. coli*
EPEC: Enteropathogenic *E. coli*
EAEC: Enteroaggregative *E. coli*
ETEC: Enterotoxigenic *E. coli*
DEC: Diarrheagenic *E. coli*
TSB: Tryptone Soya Broth
Master mix: M/m.
